# Evaluation of the Association Between lncRNA-NEAT1 and CircRNA-UHRF1 Expression Levels in the Serum of SLE Patients

**DOI:** 10.1007/s12010-026-05614-z

**Published:** 2026-03-28

**Authors:** Rehab Elsayed Marzouk, Laila Mahdi, Olfat G. Shaker, Mohammed Ali Gameil, Yasmine M. Amrousy, Mai A. El Kosaier, Reem Abdelrahman, Noha O. Shawky, Marwa Kamel

**Affiliations:** 1 Department of Medical Biochemistry and Molecular Biology, Faculty of Medicine, Capital University (Formerly Helwan University), Cairo, Egypt; 2 Department of Medical Biochemistry and Molecular Biology, Faculty of Medicine, Cairo University, Cairo, Egypt; 3 Department of Internal Medicine and Endocrinology, Faculty of Medicine, Mansoura University, Mansoura, Egypt; 4 Department of Clinical and Chemical Pathology, Faculty of Medicine, Capital University (Formerly Helwan University), Cairo, Egypt; 5 Department of Rheumatology, Rehabilitation and Physical Medicine, Faculty of Medicine, Mansoura University, Mansoura, Egypt; 6 Department of Microbiology and Immunology, Faculty of Medicine, Capital University (Formerly Helwan University), Cairo, Egypt; 7 Department of Medical Physiology, Faculty of Medicine, Capital University (Formerly Helwan University), Cairo, Egypt

**Keywords:** SLE, lncRNA-NEAT-1, circRNA-UHRF1, Autoimmune

## Abstract

Systemic lupus erythematosus (SLE) is a chronic autoimmune disorder marked by immune system dysfunction, resulting in systemic inflammation and organ damage. To measure and evaluate the association between long non-coding RNA (lncRNA)-NEAT1 and circular RNA (circRNA)-UHRF1 expression levels in the serum of SLE patients and to compare the results with healthy controls to evaluate their potential as diagnostic biomarkers regarding the disease severity. One hundred subjects subdivided equally into SLE patients and healthy-matched individuals were enrolled. Real-time PCR (RT-qPCR) for quantitative expression levels of NEAT-1 and circRNA-UHRF1 was used. The expression levels of both NEAT-1 and circRNA-UHRF1 in the serum of SLE patients were significantly upregulated in comparison with healthy controls. Results also revealed that the expression level of both NEAT-1 and circRNA-UHRF1 tended to increase with the severity of the disease. It was detected as well that there were negative correlations between circRNA-UHRF1 and TLC (*r*=-0.284, *p* = 0.046), circRNA-UHRF1 and serum albumin (*r*=-0.297, *p* = 0.036). There were positive correlations between ESR and CRP (*r* = 0.668, *p* < 0.0001), ESR and neutrophils percentage (*r* = 0.364, *p* = 0.009), ESR and urinary albumin (*r* = 0.285, *p* = 0.045), ESR and interstitial inflammation (*r* = 0.309, *p* = 0.029) and negative correlations between ESR and lymphocytes percentage (*r*=-0.315, *p* = 0.026), ESR and ALT (*r*=-0.364, *p* = 0.009). Though renal biopsy as well as laboratory tests are generally used to diagnose SLE considering symptoms, it is an invasive cost-effective procedure. The combination of evaluating NEAT-1 with circRNA-UHRF1 could be a promising non-invasive early diagnostic indicator and therapeutic target for SLE.

## Introduction

Systemic lupus erythematosus (SLE) is a chronic autoimmune disease that may result in multiple organ system damage that is diagnosed through a combination of clinical evaluation as well as laboratory testing. The severity of SLE varies from one individual to another where clinicians assess symptoms such as fatigue, joint pain, photosensitivity, butterfly-shaped skin rash or even fever [1–3].

Its prevalence varies across different regions and populations where genetic susceptibility, environmental exposures, healthcare access, and diagnostic capabilities across regions are factors affecting the disease. Globally, SLE affects approximately 3.41 million individuals, with an estimated prevalence of 43.7 cases per 100,000 individuals. As in the Middle East, the rates are lower but still significant, reporting a prevalence of 6.1 per 100,000 among adults in Egypt [4, 5].

However clinical assessment and laboratory testing are essential for the diagnosis of SLE in addition kidney biopsies are considered as definitive diagnosis for organs damages, various studies highlighted the promising role of long non coding RNAs as well as circular RNAs in the early diagnosis of autoimmune and inflammatory diseases generally or even understanding the mechanism of a disease [4–8].

Nuclear-enriched abundant transcript 1 (NEAT-1) is a long non-coding RNA (lncRNA) that plays a vital role in regulating immune responses and inflammation. It is essential for the formation of subnuclear structures (paraspeckles) that influence gene expression by sequestering transcription factors and RNA-binding proteins. In SLE, the upregulation of NEAT-1 leads to increasing immune activation and inflammation and it modulates monocyte and macrophage activation, leading to increased production of pro-inflammatory cytokines [9, 10].

Circular RNAs (circRNAs) are a type of noncoding RNAs that have a vital role in the regulation of gene expression and modulation of immune responses as well as inflammation control. Downregulation of ubiquitin-like with PHD and RING finger domains 1 (UHRF1) increases the expression of the transcription factor B cell lymphoma 6 (BCL6) by reducing DNA methylation and trimethylation of histone H3 at lysine 27 (H3K27me3), a repressive chromatin mark associated with transcriptional silencing. This process promotes T follicular helper (Tfh) cell differentiation, which plays a crucial role in the pathogenesis of systemic lupus erythematosus (SLE) [6, 11].

The actual role of circRNA-UHRF1 in autoimmune disease or inflammation has not been fully covered, however UHRF1 gene encodes a protein that regulates DNA methylation and chromatin modification [12]. Various studies highlighted the role of UHRF1 protein in immune regulation and inflammation showing its functions as an epigenetic regulator, influencing proinflammatory gene expression in rheumatoid arthritis (RA) and contributing to immune dysregulation in SLE [11, 13].

Mechanistically, NEAT-1 and UHRF1 may converge on shared epigenetic pathways regulating immune activation. NEAT-1, by modulating the nuclear localization and activity of transcription factors and chromatin regulators within paraspeckles, may indirectly influence UHRF1-mediated DNA methylation and histone modification patterns. This interaction can amplify dysregulated immune responses in SLE, leading to sustained inflammation and autoantibody production [11, 14, 15].

To our knowledge, no prior studies have simultaneously investigated NEAT-1 and circRNA-UHRF1 expression together in SLE patients, making this combined analysis a novel approach to understanding their potential as diagnostic and therapeutic biomarkers. Thus, the main purpose of the present study is to measure the association between lncRNA-NEAT1 and circRNA-UHRF1 expression levels in the serum of SLE patients and compare the results with healthy controls. In addition, this study aimed to investigate the serum biomarkers concerning the disease severity with the expectation that they may serve as diagnostic markers for SLE.

## Subjects and Methods

The sample size was calculated using the equation below, and a total of 100 individuals fulfilling the rules of the Declaration of Helsinki 1975were enrolled in this study [16]:

$$\:\mathrm{N}=\frac{{{\mathrm{Z}}_{1-\frac{}{2}}}^{2}\:\mathrm{p}(1-\mathrm{p})}{{\mathrm{d}}^{2}}=44.46$$

where the accepted margin of error was set at 5.4%. with confidence level (CI) of 95%, agreeing on 50 subjects for SLE patients’ group and another 50 for healthy controls included in the study. The threshold of significance is 0.05. Patients were recruited from the outpatient clinics or the inpatient wards of Rheumatology, Rehabilitation and Physical Medicine Department, Mansoura University hospitals. The study protocol was approved by the Faculty of Medicine, Mansoura University with approval code R.24.09.2805 on 16/10/2024. All methods were performed following the relevant guidelines and regulations. A written informed consent was obtained from each participant before the start of the study as well.

The inclusion criteria included adult subjects diagnosed with SLE according to the 2019 European League Against Rheumatism/American College of Rheumatology classification criteria for systemic lupus erythematosus [17].

Individuals with any other autoimmune diseases were excluded from the study. In addition to children, pregnant and lactating women or cognitively impaired or mentally disabled subjects were also excluded.

### Laboratory and Molecular Biology Techniques

For all participants, 3 ml venous blood sample was obtained into vacutainer tubes to determine laboratory investigation tests including Total Leukocyte Count (TLC), Erythrocyte Sedimentation Rate (ESR), C-Reactive Protein (CRP), Hemoglobin (Hb), Neutrophils, Lymphocytes, Esino and Eosinophil Percentage, Platelet Count (PLT): Creatinine, Glomerular Filtration Rate (GFR), Urea, Aspartate Aminotransferase (AST), Alanine Aminotransferase (ALT), Serum Albumin, Urinary Casts, Urinary Level, 24-Hour Protein (24-Hrs Ptn), Fasting blood glucose and Serum Uric Acid.

## Real-time Quantitative Polymerase Chain Reaction (qPCR) for the Expression of lncRNA-NEAT1 and circRNA-UHRF1

The extraction of total RNA was done using miRNeasy extraction kit (Qiagen, Valencia, CA, USA) and TRIzol reagent (Zymo Research, Irvine, CA). RNA was measured using NanoDrop2000 (Thermo Fischer Scientific, Waltham, MA). The whole RNA samples were stored at -80 °C before use. Reverse transcription was carried out on the RNA using SensiFAST cDNA Synthesis Kit [Bioline, Memphis, TN] in a final volume of 20 µl reactions. The serum relative expression level of NEAT-1 and cirUHRF1 were evaluated using GAPDH as an internal control using the Hera plus SYBR Green qPCR kit according to the manufacturer’s protocol. Fold change was calculated using the comparative threshold cycle [2^−ΔΔCt^] for relative quantification normalized to an endogenous control.

CircRNA Ubiquitin-like with PHD and RING finger domain 1 (circUHRF1) (hsa_circ_0048677) is spliced from the 12, 13 exons of UHRF1 gene and locates on chr19:4,950,622–4,951,008. CircUHRF1; Forward primer (5ʹ-3ʹ), GCTATGAGGATGATGTGGGAT, and Reverse primer (5ʹ-3ʹ), CAGAGTCTGTTCACGTCGTCC. NEAT1 primer sequences were Forward: 5’-TGGCTAGCTCAGGGCTTCAG-3’ and Reverse 5’-TCTCCTTGCCAAGCTTCCTTC-3’.

## Statistical Analysis of Data

Statistical Package of Social Science (SPSS) software version 22.0 on Windows 8.1 (SPSS Inc., Chicago, IL, USA) was used. Kruskal-Wallis test was used for the normality distribution test. Mean average ± standard deviation (SD) were used for parametric quantitative data. For qualitative data, frequencies and percentages were used. Independent Student t-test and Chi-square test (χ2) were used for parametric and non-parametric data analysis respectively. Pearson correlation (r) test was performed for correlating two quantitative variables. Receiver Operating Characteristics (ROC) curves were carried out at a significance of ≤0.05.

## Results

### Descriptive Characteristics and Laboratory Investigations of the Studied Populations

In the present study, 100 individuals subdivided into two equally matched age and sex groups (SLE and healthy controls) compared together. No significant difference was observed in age, gender ratio, and comorbidities (smoking and diabetes). A slight significance was detected as regards hypertension with *p* = 0.042. Regarding BMI in addition to systolic and diastolic blood pressure, a significant difference was detected with *p* = 0.001, 0.022, and 0.007 respectively between SLE and control. For the two groups, laboratory tests were performed including TLC, ESR, CRP, Hb, Neutrophils, Lymphocytes, Esino and Eosinophil Percentage, PLT, Creatinine, GFR, AST, ALT, Serum Albumin, Urinary Casts, Urinary Albumin Level, 24-Hour Protein, Fasting blood glucose and Serum Uric Acid. Results showed a statistical significance between the SLE group and healthy controls as regards (TLC, ESR, CRP, Neutrophils %, Lymphocytes %, PLT count, Creatinine, Urea, serum albumin, Urinary albumin, and serum uric acid) where *p* < 0.05 (Table 1).

**Table 1:** Descriptive characteristics and laboratory tests for SLE patients’ group and healthy control

Variable	SLE Patients (*N* = 50)	Healthy Control (*N* = 50)	*p*-value
Age (Yrs)	32.22±7.72	32.62±6.50	0.126
Sex (Female: Male)	42(84%): 8(16%)	42(84%): 8(16%)	1.0
Weight (kg)	77.10±14.76	-	
Height (cm)	163.76±6.47	-	
BMI	28.81±5.92	32.46±4.98	0.001*
Waist circumference (cm)	87.86±12.09	-	**-**
Systolic blood pressure	119.0±10.35	114.70±12.59	0.022*
Diastolic blood pressure	76.60±6.58	72.70±7.57	0.007*
Temperature (degree C)	36.92±0.144	-	
Age at onset (Yrs)	25.94±7.08	-	-
SLE Duration	4 (1–25)	-	-
Smoking (No: Yes)	50 (100%):0	50 (100%):0	1.0
Diabetes (No: Yes)	46 (92%):4 (8%)	50 (100%):0	1.0
HTN (No: Yes)	32 (64%):18 (36%)	50 (100%):0	0.042*
Laboratory investigation			
TLC (1000/cm3)	6.85±2.40	5.97±1.61	0.033*
ESR (mm/1st h)	37.40±18.88	40.58±25.66	0.013*
CRP (mg/dl)	3.25±3.16	1.05±0.025	0.0001*
Hb (gm/dl)TLC (1000/cm3)	11.58±1.63	10.73±1.26	0.141
Neutrophils %	61.13±10.69	9.32±4.65	0.0001*
Lymphocytes %	28.59±10.73	1.19±0.58	< 0.0001*
Eosinophil %	1.0 (0–5)	-	-
PLT count(1000/cm3)	263.04±97.81	312.12±76.90	0.006*
Creatinine (mg/dl)	0.83±0.32	0.70±0.19	0.012*
GFR (ml/min/1.73 m^2)	97.47±33.07	-	-
Urea(mg/dl)	34.82±18.97	27.34±10.54	0.017*
AST(U/L)	21.36±9.53	18.80±8.76	0.165
ALT(U/L)	20.64±9.68	17.74±9.65	0.137
s.albumin (g/dl)	3.71±0.73	3.16±0.25	0.0001*
casts(0: 4)	49 (98%): 1 (2%)	-	
Urinary albumin (0:1:2:3)	31 (62%): 8(16%): 7(14%): 4 (8)	0	< 0.0001*
24 h Ptn(g/d)	0.15 (0–9)	-	
Fasting blood glucose (mg/dl)	82.66±12.59	-	
s.uric acid(mg/dl)	4.50 (3.0–4.80)	4.20 (2.30–9.60)	0.001*

Data shown as mean±SD. Independent Student t-test was used for parametric data. SLE Duration, Esino %, 24 h Ptn, s.uric acid are shown as median (Range) are shown as Median (Range). Data shown as N (%) Chi-squared Test is used. Mann-Whitney U Test is used. *Significant at *p*≤0.05. BMI: Body mass index, HTN: hypertension, *TLC* Total Leukocyte Count, *ESR* Erythrocyte Sedimentation Rate, *CRP* C-Reactive Protein, *Hb* Hemoglobin, *PLT count* Platelet Count, *Creat* Creatinine, *GFR* Glomerular Filtration Rate, *AST* Aspartate Aminotransferase, *ALT* Alanine Aminotransferase, *s.albumin* Serum Albumin,* casts* Urinary Casts, *Urinary albumin*: Urinary Albumin Level, *24 h Ptn* 24-Hour Protein, *s.uric acid* Serum Uric Acid

### Clinical Manifestations and Complications of SLE Patients’ Group

Among the clinical features observed, 64% of the patients (32 individuals) had a fever, 38% (19) experienced fatigue, 16% (8) reported weight loss, and 74% (37) showed photosensitivity. Additionally, 50% (25) had malar rash, 10% (5) discoid rash, 52% (26) oral ulcers, 44% (22) alopecia, 10% (5) Raynaud’s phenomenon, 38% (19) arthralgia, 48% (24) arthritis, 24% (12) myalgia, 20% (10) pleurisy, 12% (6) pericardial effusion, 4% (2) myocarditis, 6% (3) endocarditis, 14% (7) vasculitis, 6% (3) mood disorders, 2% (1) transverse myelitis, 10% (5) mononeuropathy, 44% (22) leucopenia, 22% (11) thrombocytopenia, 18% (9) gastritis and 4% (2) fatty liver.

None of the patients (0%) had livedo, pneumonia, alveolar hemorrhage, shrinking lung syndrome, interstitial pulmonary fibrosis, coronary artery disease, headache, psychosis, epilepsy, acute confusional state, anxiety, cognitive dysfunction, multiple sclerosis, chorea, polyneuropathy, cranial neuropathy, Guillain-Barré syndrome, myasthenia gravis, hepatomegaly, splenomegaly, inflammatory bowel disease, mesenteric vasculitis, retinal changes, extensive scarring, scarring alopecia, significant tissue loss, bowel infarction, pancreatic insufficiency, or muscle atrophy.

For all patients, a full medical investigation was performed, showing the frequency distribution of patients as regards pattern of organ involvement, metabolic syndrome parameters, and autoimmune profile analysis (Table 2).

**Table 2:** Medical investigation of SLE patients’ group (*N* = 50)

Variable	*N* (%)	*p*-value
The pattern of organ involvement		
Constitutional (Absent: Present)	12 (24%):38(76%)	0.0001*
Mucocutaneous (Absent: Present)	6 (12%):44 (88%)	0.0001*
Musculoskeletal (Absent: Present)	7 (14%):43 (86%)	0.0001*
Pulmonary (Absent: Present)	36 (72%):14 (28%)	0.002*
Cardiac (Absent: Present)	42 (84%):8 (16%)	< 0.0001*
CNS (Absent: Present)	38 (76%):12 (24%)	0.001*
PNS (Absent: Present)	45 (90%):5 (10%)	< 0.0001*
Hematological (Absent: Present)	22 (44%):28 (56%)	0.396*
GIT (Absent: Present)	46 (92%):4 (8%)	< 0.0001*
Vasculitis (Absent: Present)	42 (84%):8 (16%)	< 0.0001*
Ocular (Absent: Present)	47 (94%):3 (6%)	< 0.0001*
Venous/arterial thrombosis (Absent: Present)	41 (82%):9 (18%)	< 0.0001*
APS (Absent: Present)	34 (68%):16 (32%)	0.011*
Metabolic syndrome parameters		
Fasting blood glucose (FBG) ≥ 100 m/dl (No: Yes)	49 (98%):1 (2%)	< 0.0001*
Receiving anti-hyperglycemic drug (No: Yes)	46 (92%):4 (8%)	< 0.0001*
Blood pressure (BP) ≥ 130/85 mmHg or receiving hypertensive drug (No: Yes)	31 (62%):19 (38%)	0.090
Triglycerides (TG) ≥ 150 mg/dl	30 (60%):20 (40%)	0.157
High-density lipoproteins-cholesterol (HDL-C) < 40 mg/dl (male), < 50 mg/dl (female) (No: Yes)	24 (48%):26 (52%)	0.777
Waist circumference (WC) ≥ 102 cm (male), ≥ 88 cm (female) (No: Yes)	27 (54%):23 (46%)	0.572
Autoimmune profile		
Antinuclear antibody (ANA) pattern		
Speckled	35 (70%)	< 0.0001*
Homogenous	13 (26%)	
Cytoplasmic	1 (2%)	
Rim	1 (2%)	
antidsDNA ab (Negative: Positive)	18 (36%): 32 (64%)	0.048*
C3 (Normal: Consumed)	42 (84%):8 (16%)	0.0001*
C4 (Normal: Consumed)	45 (90%):5 (10%)	< 0.0001*
Lipid profile Analysis		
Diagnosis of dyslipidemia (Absent: Present)	17 (34%): 33 (66%)	0.011*
Total cholesterol (TC) mg/dl	200.28±45.45	**-**
High-density lipoproteins-cholesterol (HDL-C) (mg/dl)	47.36±10.08	**-**
Low-density lipoproteins-cholesterol (LDL-C) (mg/dl)	119.22±43.01	**-**
Triglycerides (TG) mg/dl	141.92±54.94	**-**

Data shown as N (%) Chi-squared Test was used. Mann-Whitney U Test for significance. Lipid profile analysis is shown as mean±SD. Independent Student t-test was used. *CNS* Central Nervous System, *PNS* Peripheral Nervous System, *GIT*: Gastrointestinal Tract, *APS *Antiphospholipid Syndrome, *RIM* Rim pattern, anti-dsDNA, *Ab* Anti-double-stranded DNA Antibody, *C3/C4* Complement Component 3/4. * Significant at *p* ≤0.05

## Renal Biopsy and Medication Intake for SLE Patients’ Group

A biopsy was taken from each patient and analyzed for glomeruli percentage monitoring various variables including Endocapillary hypercellularity, Leukocyte infiltration, Subendothelial hyaline deposits, Fibrinoid necrosis, Cellular crescents, Interstitial inflammation, Glomerular sclerosis, Fibrous crescents, Tubular atrophy and Interstitial fibrosis where accordingly patients were classified based on the percentage (0 = not present, 1 = < 25% of glomeruli, 2 = 25%-50% of glomeruli, 3 = > 50% of glomeruli). Results revealed that concerning the variables in renal biopsy, there was a high statistical significance detected between glomeruli percentage variables with *p* < 0.0001 for all variables (Table 3).

**Table 3:** Renal biopsy results of SLE patients’ group (*N* = 50)

Variables					
% of glomeruli	Not Present	< 25%	25%-50%	> 50%	*p*-value
Endocapillary hypercellularity	36 (72%)	4 (8%)	3 (6%)	7 (14%)	< 0.0001*
Leukocyte infiltration	37 (74%)	3 (6%)	4 (8%)	6 (12%)	< 0.0001*
Subendothelial hyaline deposits	46 (92%)	1 (2%)	0	3 (6%)	< 0.0001*
Fibrinoid necrosis	46 (92%)	1 (2%)	1 (2%)	2 (4%)	< 0.0001*
Cellular crescents	45 (90%)	3 (6%)	1 (2%)	1 (2%)	< 0.0001*
Interstitial inflammation	38 (76%)	2 (4%)	1 (2%)	9 (18%)	< 0.0001*
Glomerular sclerosis	36 (72%)	10 (20%)	4 (8%)	0	< 0.0001*
Fibrous crescents	50 (100%)	0	0	0	< 0.0001*
Tubular atrophy	49 (98%)	0	0	1 (2%)	< 0.0001*
Interstitial fibrosis	44 (88%)	4 (8%)	1 (2%)	1 (2%)	< 0.0001*

Data shown as N (%) Chi-squared Test was used. Mann-Whitney U Test for significance. 0 = not present, 1 = < 25% of glomeruli, 2 = 25%-50% of glomeruli, 3 = > 50% of glomeruli. * Significant at *p* ≤0.0.5

Of the medical treatment and intake, Steroids, Cyclophosphamide (CYC), Hydroxychloroquine (HCQ), Azathioprine (AZA), Mycophenolate mofetil (MMF), Cyclosporine A (CSA), Rituximab, Angiotensin-Converting Enzyme Inhibitors (ACEi) and Angiotensin II Receptor Blockers (ARBs) were included. The median dose of steroid was 11.06 mg with a range of 5–25 mg with a duration of median of 3.19 months for all patients. The cumulative CYC dose was 0 (0–12 mg). For HCQ, AZA, MMF, CSA, Rituximab, ACEi, and ARBs the median of the doses was 200 (0–400 mg), 0 (0–150 mg), 0 (0–2gm), 0 (0–100 mg) respectively, and for Rituximab, ACEi and ARBs it is 0 (0–1) for each,

### The Expression Levels of lncRNA-NEAT-1 and circRNA-UHRF1 Fold Changes in Serum for SLE and Healthy Control Groups

**Fig. 1:**
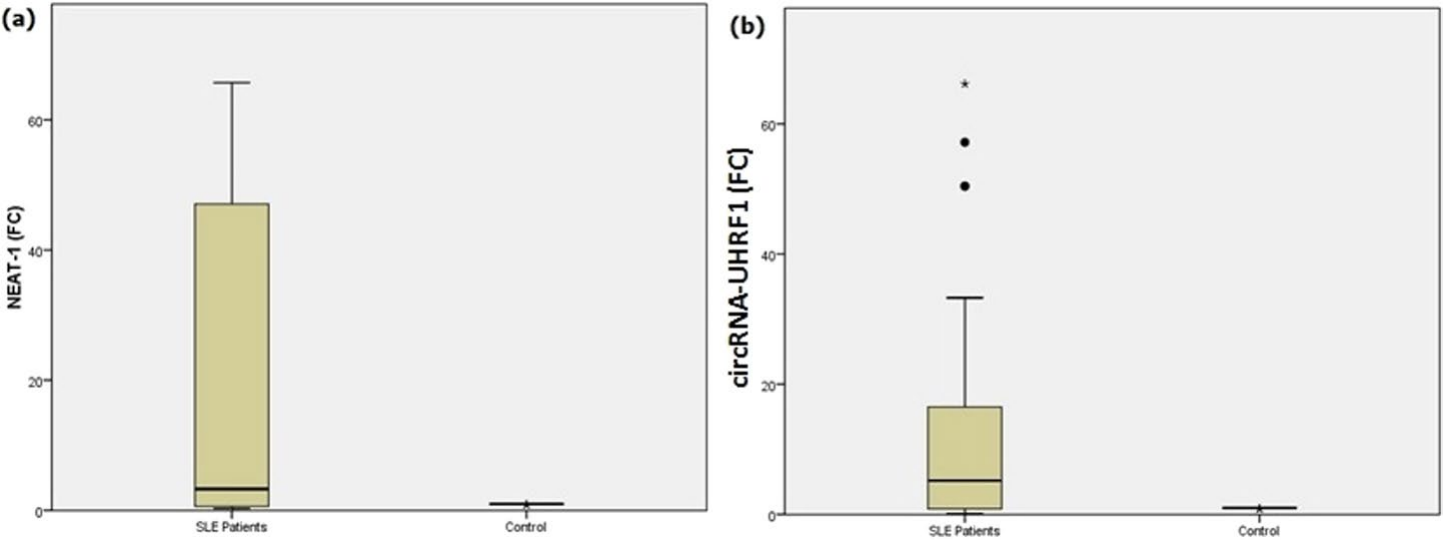
The expression levels of serum biomarkers for SLE patients and healthy control groups (**a**) lncRNA-NEAT1 (**b**) circRNA-UHRF1 fold changes

For both groups, the expression level of NEAT-1 and circRNA-UHRF1 fold changes was measured and compared accordingly with healthy controls. Results revealed that a high significant difference was detected between SLE patients with median and Interquartile range (IQR) of 3.28 (0.30-65.39) for NEAT-1 and 5.19 (0.15–65.13) for circRNA-UHRF1 compared to [1] for controls with *p* < 0.0001 for each (Fig. 1).

## Association of lncRNA-NEAT-1 and CircRNA-UHRF1 with Disease Severity

Based on the Systemic Lupus Erythematosus Disease Activity Index 2000 (SLEDAI-2 K), patients were classified into inactive (SLEDAI = 0), mild activity (SLEDAI = 1 to 5), moderate activity (SLEDAI = 6 to 10), high activity (SLEDAI = 11 to 19), and very high activity (SLEDAI≥20). Patients were classified into inactive SLE, mild, and moderate where results revealed that the expression levels of both NEAT-1 and circRNA-UHRF1 tended to increase with the severity of the disease (Table 4).

**Table 4:** Serum levels of lncRNA-NEAT1 and circRNA-UHRF1 fold changes as regards disease activity based on SLEDAI-2K and SLICC DI patient's score

SLEDAI-2K					
Serum Biomarkers	Inactive	Mild Activity	Moderate Activity		*p*-value
lncRNA-NEAT1 (FC)	**1.51**(0.30-57.56)	**9.73**(0.52-57.56)	**52.90**(20.7-65.70)		0.37^x^, **0.049**^**y**^, 0.12^z^
CircRNA-UHRF1(FC)	**5.19**(0.14-66.13)	**9.54**(0.36-57.17)	**12.34**(0.96-32.81)		0.80^x^, 0.32^y^, 0.59^z^
SLICC DI					
Serum Biomarkers	0 (*N*=34)	1 (*N*=10)	2 (*N*=1)	3 (*N*=5)	*p*-value
lncRNA-NEAT1 (FC)	**9.71**(0.30-65.70)	**1.52**(0.52-57.56)	**0.59**	**0.49**(0.34-57.5)	0.65^a^, 0.23^b^, 0.25^c^, 0.36^d^, 0.32^e^, 0.77^f^
CircRNA-UHRF1 (FC)	**5.19**(0.26-50.43)	**7.52**(0.14-57.17)	**16.52**	**0.85**(0.85-66.13)	0.72^a^, 0.29^b^, 0.83^c^, 0.52^d^, 0.62^e^, 0.35^f^

Data shown as median (Range), Chi-squared Test is used. SLEDAI-2K: Systemic Lupus Erythematosus Disease Activity Index 2000, SLICC DI: Systemic Lupus International Collaborating Clinics Damage Index. Inactive (SLEDAI =0), mild activity (SLEDAI=1 to 5), moderate activity (SLEDAI=6 to 10), high activity (SLEDAI=11 to 19), and very high activity (SLEDAI ≥ 20). x between (Inactive and Mild activity), y between (Inactive and Moderate activity), z between (Mild activity and Moderate activity). a between 0 and 1, b between 0 and 2, c between 0 and 3, d between 1 and 2, e between 1 and 3, f between 2 and 3. Mann-Whitney U Test for significance. *Significant at *p* ≤ 0.05

### Correlation between Different Parameters and the Study Serum Biomarkers of SLE Patients’ Group

**Fig. 2:**
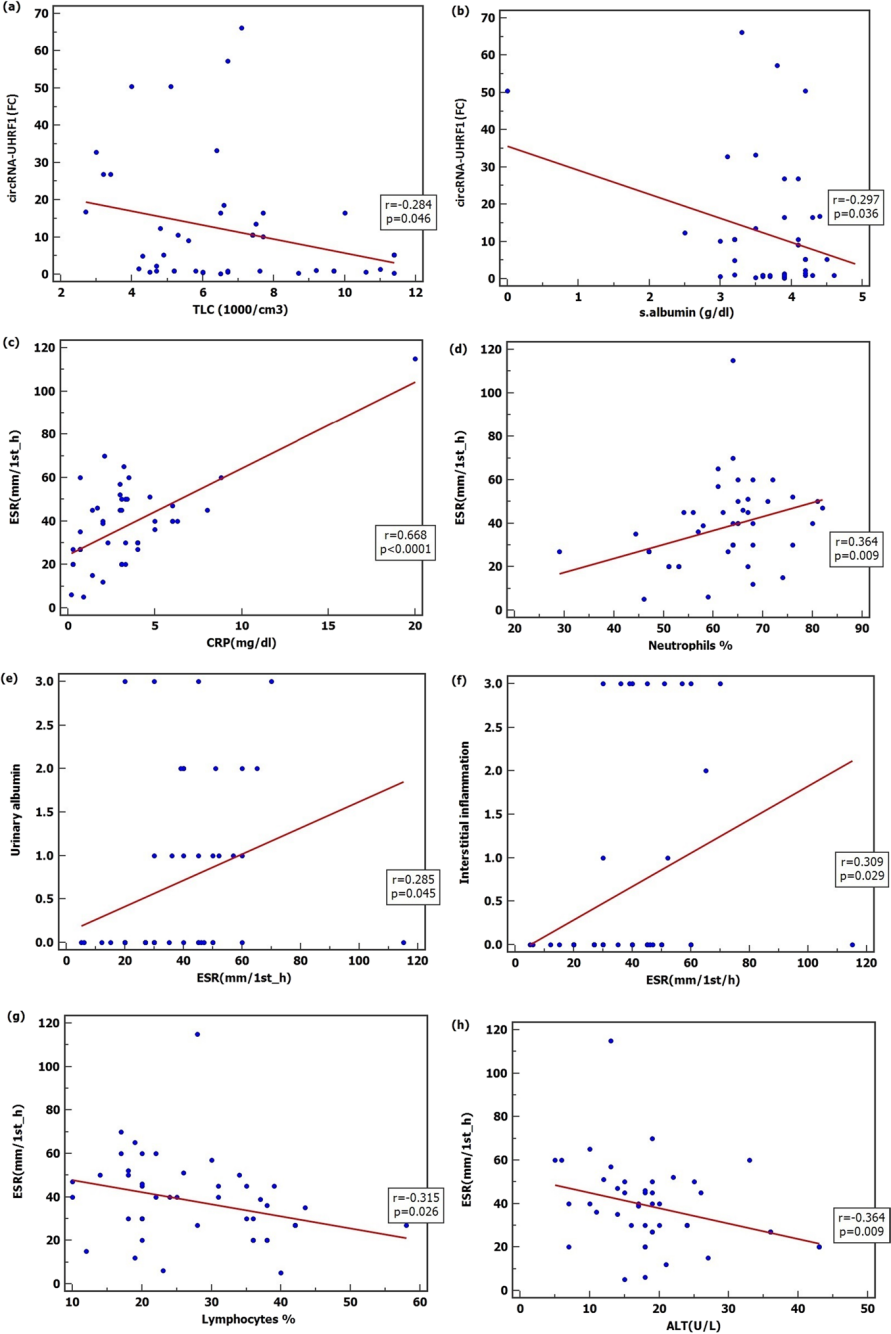
Correlation between serum biomarkers of SLE patients’ group and laboratory data (**a**) circRNA-UHRF1 and Total Leukocyte Count (TLC) (**b**) circRNA-UHRF1 and serum albumin (s. albumin) (**c**) Erythrocyte Sedimentation Rate (ESR) and C-reactive protein (CRP) (**d**) ESR and Neutrophils percentage (**e**) Erythrocyte Sedimentation Rate (ESR) and Urinary albumin (**f**) Erythrocyte Sedimentation Rate (ESR) and Interstitial inflammation (**g**) Erythrocyte Sedimentation Rate (ESR) and Lymphocyte percentage (h) Erythrocyte Sedimentation Rate (ESR) and Alanine Aminotransferase (ALT)

According to Pearson correlation, results showed that there were negative correlations between circRNA-UHRF1 and TLC (*r*=-0.284, *p* = 0.046), circRNA-UHRF1 and serum albumin (*r*=-0.297, *p* = 0.036). There were positive correlations between ESR and CRP (*r* = 0.668, *p* < 0.0001), ESR and neutrophils percentage (*r* = 0.364, *p* = 0.009), ESR and urinary albumin (*r* = 0.285, *p* = 0.045), ESR and interstitial inflammation (*r* = 0.309, *p* = 0.029) and negative correlations between ESR and lymphocyte percentage (*r*=-0.315, *p* = 0.026), ESR and ALT (*r*=-0.364, *p* = 0.009) (Fig. 2).

### Sensitivity and Specificity of lncRNA-NEAT-1 and circRNA-UHRF1 Serum Biomarkers among SLE Patients’ Group

**Fig. 3:**
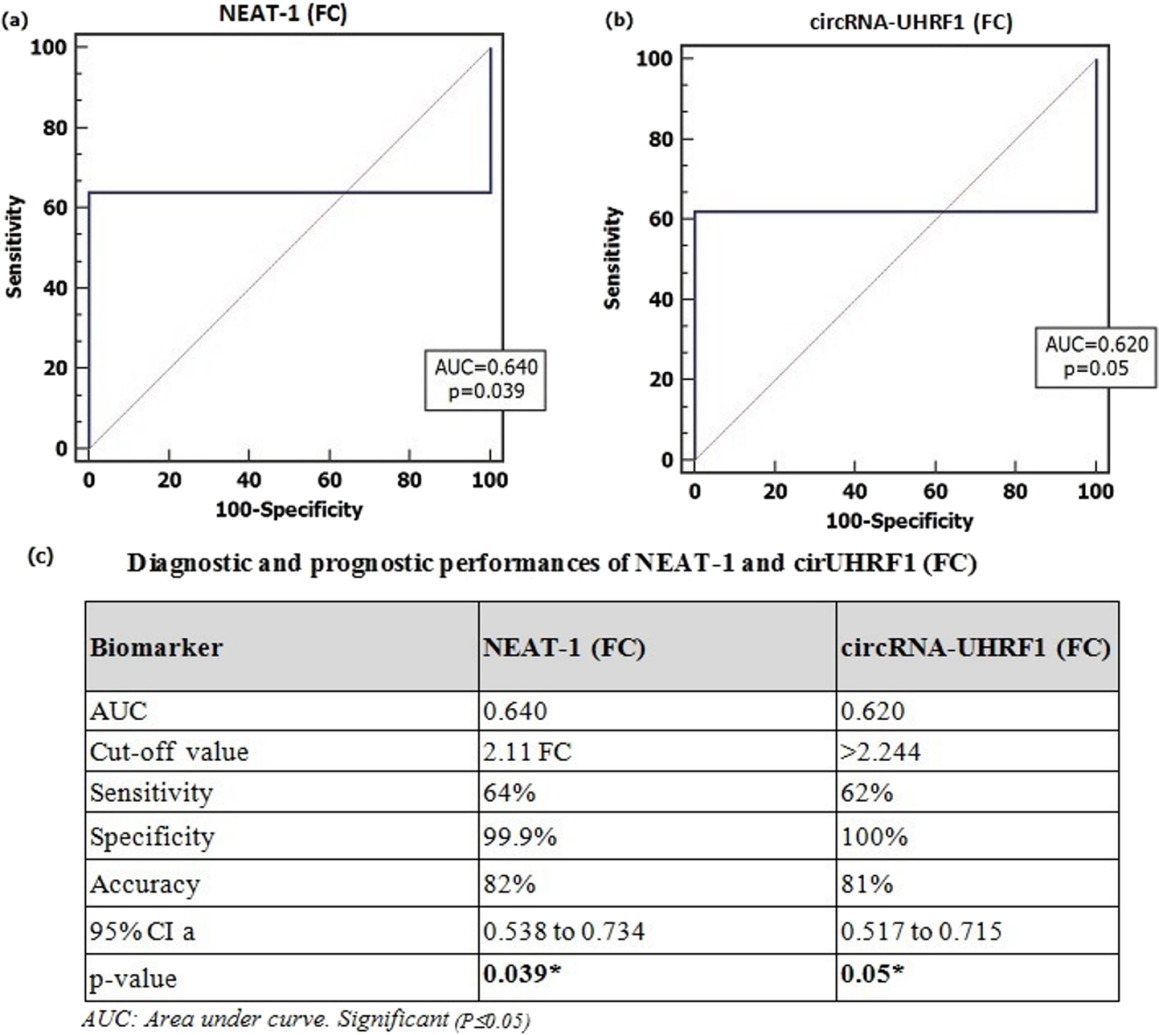
ROC curve analysis for the study serum biomarker among the studied groups (**a**) ROC curve for lncRNA-NEAT1 among SLE patients versus healthy controls (**b**) ROC curve for circRNA-UHRF1 among SLE patients versus healthy controls (**c**) Diagnostic and prognostic performances of lncRNA-NEAT1 and circRNA-UHRF1 (FC) among SLE patients’ group

According to the calculation of the results of the expression levels of lncRNA-NEAT1 and circRNA-UHRF1 fold changes in serum samples of SLE patients, the applied values of the biomarkers were determined as indicators for the diagnosis of patients. Receiver Operating Characteristics (ROC) curves were carried out for analysis showing the area under curve (AUC) was 0.640 and 0.620 for lncRNA-NEAT1 and circRNA-UHRF1 with sensitivity and specificity of 64%; 99.9% for lncRNA-NEAT1 and 62% and 100% for circRNA-UHRF1 respectively (Fig. 3).

## Discussion

In the present study, we evaluated the expression levels of lncRNA-NEAT-1 and circRNA-UHRF1 fold changes in the serum of SLE patients and healthy controls. Results revealed that both biomarkers were upregulated in SLE patients’ groups compared to controls.

In addition, when measuring the serum biomarkers concerning the disease severity, it was detected that lncRNA-NEAT1 and circRNA-UHRF1 tended to elevate with the severity of the disease activity showing significant differences between patients with mild, moderate, and inactive SLE patients.

In the present study, our findings revealed statistically significant differences between the SLE patients’ group and the control regarding body mass index, systolic and diastolic blood pressure, and patients with hypertension. In addition, TLC, neutrophils, lymphocyte percentages, creatinine levels, albumin levels, and uric acid levels in serum tended to be higher in the patients’ group than in controls. While ESR was reported lower in the patients’ group.

In agreement with our findings, various studies have investigated the expression levels of lncRNA-NEAT1 in SLE patients pointing to its potential role in its pathogenesis. The level of lncRNA-NEAT1 in peripheral blood mononuclear cells (PBMCs) of SLE patients was upregulated when compared to controls suggesting that lncRNA-NEAT1 influences the expression of inflammatory chemokines and cytokines by activating the mitogen-activated protein kinase (MAPK) signaling pathway, contributing to SLE pathogenesis [18].

Similar to our findings, a study reported that elevated lncRNA-NEAT1 levels in monocyte-derived dendritic cells (moDCs) of SLE patients, indicated a positive correlation with disease activity. The study found that lncRNA-NEAT1 acts as a competitive endogenous RNA, sponging miR-365a-3p and promoting interleukin-6 (IL-6) secretion, thereby enhancing the inflammatory response in SLE [15, 19].

Studies also identified the role of lncRNA-NEAT1 by which the increased levels indicate that increased expression of NEAT-1 correlates positively with disease activity and regulates cytokine and chemokine production through the MAPK pathway, suggesting its potential as a therapeutic target in SLE [20].

In the present study, negative correlations between circRNA-UHRF1 and TLC, circRNA-UHRF1 and serum albumin, ESR and lymphocyte percentage, ESR and ALT were detected and positive correlations between ESR and CRP, ESR and neutrophils percentage, ESR and urinary albumin, ESR and interstitial inflammation were also reported.

The correlation between circRNA-UHRF1 and total leukocyte count (TLC) has not been discussed previously. However, circRNA-UHRF1 has a vital role in cancer, especially hepatocellular carcinoma (HCC), where it has been shown to impair natural killer (NK) cell function and contribute to immune evasion [21].

To our knowledge, no previous studies have addressed the association between circRNA-UHRF1 and TLC.Although certain circular RNAs have been implicated in autoimmune diseases, the specific role of circRNA-UHRF1 remains underexplored as no direct evidence linking circRNA-UHRF1 to variations in leukocyte counts.

As well, there is limited research directly examining the correlation between ESR and ALT levels in SLE or other autoimmune and inflammatory diseases. ESR is considered a nonspecific marker of inflammation and ALT is an enzyme indicative of hepatocellular injury. In autoimmune diseases, elevated ESR levels are commonly observed due to chronic inflammation in which the immune system attacks the body’s tissues leading to increased production of proteins that cause red blood cells to settle more quickly. In SLE, the correlation can be weak or inconsistent due to the influence of type I interferons [22].

In the present study, results revealed that ESR levels are slightly lower than healthy control however studies have demonstrated that changes in ESR correlate with disease activity indices in SLE, such as the physician global assessment (PGA), renal involvement, fatigue, and joint visual analog scale (VAS) scores. No studies directly assessed the relationship between ESR and ALT levels in SLE [23].

Similarly, the direct correlation between ESR and ALT was not explored in SLE. However, in other autoimmune conditions like Sjögren’s syndrome, elevated ALT levels have been associated with a grimmer prognosis [24].

Regarding the relationship between ESR and CRP levels in SLE, no direct studies demonstrated it. However, both have been considered as markers of inflammation but their behaviors differ in SLE patients [25]. While the level of CRP in SLE patients do not always reflect disease activity. In general, it rises markedly in response to bacterial infections or acute inflammatory conditions. However, during SLE flares, CRP levels may remain low or only modestly elevated, likely due to the inhibitory effect of type I interferons [26].

So generally, ESR and CRP usually have a positive correlation in general inflammatory conditions. Agreeing with our findings, studies have shown the correlation between ESR and neutrophil percentage in SLE is generally positive as both markers reflect systemic inflammation where a higher neutrophil count is often observed in active inflammation, while ESR rises due to increased fibrinogen and other acute-phase reactants [27, 28].

In agreement with our findings, a study reported a positive correlation between ESR and interstitial inflammation stating that patients with drug-induced acute tubulointerstitial nephritis lupus with higher ESR levels were significantly associated with increased renal interstitial inflammatory cell infiltration, concluding that elevated ESR reflects active interstitial inflammation in the kidneys [29].

Another study discussed the role of circRNA-UHRF1 and concluded that it has a vital and promising role in the pathogenesis of SLE as the downregulation of it leads to increased transcription factor B cell lymphoma 6 (BCL6) expression by decreasing DNA methylation and H3K27me3 levels, promoting T follicular helper (Tfh) cell differentiation of SLE [11].

Circular RNA UHRF1 (circUHRF1) has been implicated in other diseases than SLE such as cancer by which findings highlighted the role of circUHRF1 in modulating tumor immunity and progression, suggesting its potential role as a therapeutic target in cancer treatment. In HCC, circUHRF1 was secreted via exosomes and tended to be overexpressed leading to natural killer (NK) cell dysfunction by upregulating TIM-3 through miR-449c-5p degradation. This contributes to immune evasion and resistance to anti-PD1 therapy [30].

In a study on pancreatic ductal adenocarcinoma patients (PDAC), circUHRF1 promotes tumor progression by sponging miR-1306-5p, resulting in the upregulation of ARL4C thus enhancing cell proliferation, migration, and epithelial-mesenchymal transition (EMT), thus facilitating tumor growth [31].

In the present study, ROC curve analysis revealed AUC values of 0.640 for lncRNA-NEAT1 and 0.620 for circRNA-UHRF1. Based on established interpretation criteria, AUC values in the range of 0.6–0.7 reflect a moderate discriminatory capacity for binary classification models. While these values do not indicate high accuracy, they still demonstrate that both NEAT1 and UHRF1 have a consistent and measurable ability to differentiate SLE patients from healthy controls, supporting their potential relevance in the disease context.

To the best of our knowledge, the combination of lncRNA-NEAT1 and circRNA-UHRF1 was not studied in SLE, making this the first investigation to evaluate their combined potential as biomarkers in this disease. Generally, circRNAs are considered an important epigenetic modulator of gene expression in inflammation and autoimmune regulation, which is closely related to the pathogenesis of other autoimmune diseases like RA [32, 33].

Although lncRNA-NEAT1 and circRNA-UHRF1 have not been jointly studied in SLE, their roles have been explored separately in other autoimmune and inflammatory disorders, which provides indirect context for our findings. A study by Chen et al. (2023) demonstrated that NEAT-1 modulates macrophage activation in RA, while other studies have linked UHRF1 dysregulation to immune imbalance in diseases such as multiple sclerosis (MS) and autoimmune hepatitis as well. These parallels suggest that both NEAT1-mediated transcriptional regulation and UHRF1-driven epigenetic control may converge on common inflammatory pathways, potentially explaining their concurrent upregulation in the present study [34, 35].

## Conclusions

In conclusion, serum expression levels of lncRNA-NEAT1 and circRNA-UHRF1 were significantly upregulated in SLE patients and showed a positive correlation with disease severity, suggesting their potential as diagnostic and therapeutic biomarkers for SLE. These findings enhance the understanding of the role of non-coding RNAs in the pathogenesis of autoimmune diseases and highlight their value as non-invasive indicators for disease monitoring and evaluation.Future investigations with larger, well-characterized cohorts are needed to validate these results, clarify their associations with specific clinical parameters, and disease activity, and evaluate their integration into diagnostic models or targeted therapeutic approaches.

## Data Availability

The data that support the findings of this study are not included in the manuscript for ethical reasons but are available upon reasonable request from the corresponding author, Olfat Shaker [email: olfat.shaker@kasralainy.edu.eg].

## References

[1] Fanouriakis, A., Kostopoulou, M., Andersen, J., Aringer, M., Arnaud, L., Bae, S. C., Boletis, J., Bruce, I. N., Cervera, R., Doria, A., Dörner, T., Furie, R. A., Gladman, D. D., Houssiau, F. A., Inês, L. S., Jayne, D., Kouloumas, M., Kovács, L., Mok, C. C., Morand, E. F., Moroni, G., Mosca, M., Mucke, J., Mukhtyar, C. B., Nagy, G., Navarra, S., Parodis, I., Pego-Reigosa, J. M., Petri, M., Pons-Estel, B. A., Schneider, M., Smolen, J. S., Svenungsson, E., Tanaka, Y., Tektonidou, M. G., Teng, Y. K. O., Tincani, A., Vital, E. M., van Vollenhoven, R. F., Wincup, C., Bertsias, G., & Boumpas, D. T. (2024). EULAR recommendations for the management of systemic lupus erythematosus: 2023 update. *Annals of the Rheumatic Diseases*, *83*(1), 15–29. 37827694 10.1136/ard-2023-224762

[2] Menyawi, M. E., Fawzy, M., Habib, M., & Shaker, O. (2018). Serum transforming growth Factor-Beta 1 level in Egyptian systemic lupus erythematosus patients. *Archives of Rheumatology*, *33*(3), 358–366. 30632531 10.5606/ArchRheumatol.2018.6405 PMC6328217

[3] Barber, M. R. W., & Clarke, A. E. (2020). Systemic lupus erythematosus and risk of infection. *Expert Review of Clinical Immunology*, *16*(5), 527–538. 32478627 10.1080/1744666X.2020.1763793

[4] Abdel-Nasser, A. M., Gabalawy, E., H. S., & Al-Bishri, J. (2021). Adult systemic lupus erythematosus in egypt: The nation-wide spectrum of 3661 patients and world-wide characteristics. *Lupus*, *30*(8), 1305–1313. 10.1177/09612033211014253 33951965

[5] Eesa, N. N., Nabi, A., Owaidy, H., Khalifa, R. E., Radwan, I., NourEl-Din, A. R., Amer, A. M., ElShereef, M. A., Hassan, R. R., Ismail, E., El-Gazzar, F., Khalil, I. I., Moshrif, N. M., Abualfadl, A. H., Tharwat, E., Fathi, S., Elazeem, H. M. A., El-Shebini, M. I., Samy, E., Noshy, N., & Egyptian College of Rheumatology (ECR) SLE Study Group. (2021). Systemic lupus erythematosus children in Egypt: Homeland spectrum amid the global situation. *Lupus*, *30*(13), 2135–2143. 34528835 10.1177/09612033211043010

[6] ElFeky, D. S., Omar, N. M., Shaker, O. G., Abdelrahman, W., Gheita, T. A., & Nada, M. G. (2024). Circulatory MicroRNAs and Proinflammatory cytokines as predictors of lupus nephritis. *Frontiers in Immunology*, *15*, 1449296. 39464895 10.3389/fimmu.2024.1449296 PMC11502402

[7] Aringer, M. (2020). Inflammatory markers in systemic lupus erythematosus. *Journal of Autoimmunity*, *110*, 102374. 31812331 10.1016/j.jaut.2019.102374

[8] El-Gamal, R., Zalata, A., Mazroa, S. A., Comhaire, F., Gamal, A., Shaker, O. G., & Hazem, N. M. (2024). Evaluation of circANKLE2 & circL3MBTL4 -RNAs expression in fertile and infertile men. *Biochemical Genetics*. 10.1007/s10528-024-10963-7Advance online publication. 39580773 10.1007/s10528-024-10963-7

[9] Mohammed, A., Shaker, O. G., Khalil, M. A. F., Elsabagh, Y. A., Gomaa, M., Ahmed, A. M., & Erfan, R. (2022). Association of long non-coding RNAs NEAT1, and MALAT1 expression and pathogenesis of Behçet’s disease among Egyptian patients. *Saudi Journal of Biological Sciences*, *29*(8), 103344. 35800145 10.1016/j.sjbs.2022.103344 PMC9253411

[10] Zhang, X., Liu, L., Zhao, J., & Li, Y. (2023). The role of long non-coding RNA NEAT1 in immune regulation and systemic lupus erythematosus. *Autoimmunity Reviews*, *22*(1), 103212.36252931

[11] Liu, L., Hu, L., Yang, L., Jia, S., Du, P., Min, X., Wu, J., Wu, H., Long, H., Lu, Q., & Zhao, M. (2021). UHRF1 downregulation promotes T follicular helper cell differentiation by increasing BCL6 expression in SLE. *Clinical Epigenetics*, *13*(1), 31. 33568199 10.1186/s13148-021-01007-7 PMC7874639

[12] Bostick, M., Kim, J. K., Estève, P. O., Clark, A., Pradhan, S., & Jacobsen, S. E. (2007). UHRF1 plays a role in maintaining DNA methylation in mammalian cells. *Science (New York)*, *317*(5845), 1760–1764. 10.1126/science.1147939 17673620

[13] Saeki, N., Inoue, K., Ideta-Otsuka, M., Watamori, K., Mizuki, S., Takenaka, K., Igarashi, K., Miura, H., Takeda, S., & Imai, Y. (2022). Epigenetic regulator UHRF1 orchestrates Proinflammatory gene expression in rheumatoid arthritis in a suppressive manner. *The Journal of Clinical Investigation*, *132*(11), e150533. 35472067 10.1172/JCI150533 PMC9151705

[14] Zhang, R. X., Zhang, Z. X., Zhao, X. Y., Liu, Y. H., Zhang, X. M., Han, Q., & Wang, X. Y. (2025). Mechanism of action of lncRNA-NEAT1 in immune diseases. *Frontiers in Genetics*, *16*, 1501115. 40110044 10.3389/fgene.2025.1501115 PMC11919857

[15] Zhang, Y., Zhang, Y., Li, Y., & Zhu, X. (2023). LncRNA NEAT1 promotes IL-6 secretion in monocyte-derived dendritic cells of systemic lupus erythematosus by sponging miR-365a-3p. *RNA Biology*, *20*(1), 1–11. 37343193 10.1080/15592294.2023.2226492 PMC10286691

[16] Charan, J., & Biswas, T. (2013). How to calculate sample size for different study designs in medical research? *Indian Journal of Psychological Medicine*, *35*(2), 121–126. 24049221 10.4103/0253-7176.116232 PMC3775042

[17] Aringer, M., Costenbader, K., Daikh, D., Brinks, R., Mosca, M., Ramsey-Goldman, R., Smolen, J. S., Wofsy, D., Boumpas, D. T., Kamen, D. L., Jayne, D., Cervera, R., Costedoat-Chalumeau, N., Diamond, B., Gladman, D. D., Hahn, B., Hiepe, F., Jacobsen, S., Khanna, D., Lerstrøm, K., Massarotti, E., McCune, J., Ruiz-Irastorza, G., Sanchez-Guerrero, J., Schneider, M., Urowitz, M., Bertsias, G., Hoyer, B. F., Leuchten, N., Tani, C., Tedeschi, S. K., Touma, Z., Schmajuk, G., Anić, B., Assan, F., Chan, T. M., Clarke, A. E., Crow, M. K., Czirják, L., Doria, A., Graninger, W., Halda-Kiss, B., Hasni, S., Izmirly, P. M., Jung, M., Kumánovics, G., Mariette, X., Padjen, I., Pego-Reigosa, J. M., Romero-Díaz, J., Rúa-Figueroa Fernández, Í., Seror, R., Stummvoll, G. H., Tanaka, Y., Tektonidou, M. G., Vasconcelos, C., Vital, E. M., Wallace, D. J., Yavuz, S., Meroni, P. L., Fritzler, M. J., Naden, R., Dörner, T., & Johnson, S. R. (2019). 2019 European League Against Rheumatism/American College of Rheumatology classification criteria for systemic lupus erythematosus. *Annals of the Rheumatic Diseases*, *78*(9), 1151–1159. 10.1136/annrheumdis-2018-214819 31383717

[18] Wu, H., Chen, S., Li, A., Shen, K., Wang, S., Wang, S., Wu, P., Luo, W., & Pan, Q. (2021). LncRNA expression profiles in systemic lupus erythematosus and rheumatoid arthritis: Emerging biomarkers and therapeutic targets. *Frontiers in Immunology*, *12*, 792884. 35003113 10.3389/fimmu.2021.792884 PMC8732359

[19] Gao, Y., Li, S., Zhang, Z., Yu, X., & Zheng, J. (2018). The role of long Non-coding RNAs in the pathogenesis of RA, SLE, and SS. *Frontiers in Medicine*, *5*, 193. 30018955 10.3389/fmed.2018.00193 PMC6038710

[20] Tang, Y., Luo, X., Cui, H., Ni, X., Yuan, M., Guo, Y., Huang, X., Zhou, H., & Lu, Q. (2017). *I*dentification of the long noncoding RNA NEAT1 as a novel inflammatory regulator acting through MAPK pathway in human lupus. *Arthritis & Rheumatology, 69*(S10). 10.1016/j.jaut.2016.07.012 27481557

[21] Zhang, P. F., Gao, C., Huang, X. Y., Lu, J. C., Guo, X. J., Shi, G. M., Cai, J. B., & Ke, A. W. (2020). Cancer cell-derived Exosomal circUHRF1 induces natural killer cell exhaustion and May cause resistance to anti-PD1 therapy in hepatocellular carcinoma. *Molecular Cancer*, *19*(1), 110. 32593303 10.1186/s12943-020-01222-5 PMC7320583

[22] Medical Outline. (2021). Why ESR is high in autoimmune disease? Retrieved from https://www.medicaloutline.com/faq/why-esr-is-high-in-autoimmune-disease/

[23] Stojan, G., Fang, H., Magder, L., & Petri, M. (2012). Erythrocyte sedimentation rate is a predictor of renal and overall SLE disease activity. *Annals of the Rheumatic Diseases*, *71*(Suppl 3), 2152. 10.1177/0961203313492578 PMC3703841 23761098

[24] Liang, P., Huang, Y., Hu, Z., Zhou, L., Cai, S., Zhong, J., & Dong, L. (2025). Clinical and laboratory characteristics of Sjögren’s syndrome-associated autoimmune liver disease: A real-world, 10-year retrospective study. *Clinical Rheumatology*, *44*(3), 1225–1236. 39826046 10.1007/s10067-024-07273-z

[25] Kim, H. A., Jeon, J. Y., Song, Y. W., & Park, J. H. (2015). The correlation between inflammatory markers and disease activity in systemic lupus erythematosus. *Annals of the Rheumatic Diseases*, *74*(Suppl 2), 1086.

[26] Lauwerys, B. R., Ducreux, J., & Houssiau, F. A. (2020). Type I interferon and systemic lupus erythematosus: Conundrums and perspectives. *Frontiers in Immunology*, *11*, 622326.33584722

[27] Mohamed, A. A., El-Gazzar, N. M., & El-Shazly, R. M. (2023). The neutrophil-to-lymphocyte ratio and systemic inflammation in autoimmune diseases. *Egyptian Rheumatology and Rehabilitation*, *50*(1), 22–30.

[28] Ali, R. S., Hassan, M. K., & Saleh, W. A. (2024). Neutrophil-to-lymphocyte ratio as an inflammatory marker in autoimmune diseases. *International Journal of Immunopathology and Pharmacology*, *38*(2), 112–125.

[29] Wang, Y., Li, Y., & Zhang, X. (2020). The role of inflammatory markers in drug-induced acute tubulointerstitial nephritis. *BMC Nephrology*, *21*(1), 175. 33243164 10.1186/s12882-020-02175-z PMC7689990

[30] Chen, L., Nan, A., Zhang, N., Jia, Y., Li, X., Ling, Y., Dai, J., & Jiang, Y. (2020). Exosomal circrna UHRF1 regulates natural killer cell function and immunoevasion by upregulating TIM-3 in hepatocellular carcinoma. *Molecular Cancer*, *19*(1), 1–19.31901224

[31] Xu, J., Ji, L., Liang, Y., Wan, Z., Zheng, W., Song, X., Geng, P., & Cheng, Y. (2021). CircUHRF1 drives pancreatic cancer progression via the miR-1306-5p/ARL4C axis. *Cancer Biology & Therapy*, *22*(1), 22–33.

[32] Ghafouri-Fard, S., Shoorei, H., Sabernia, T., Hussen, B. M., Taheri, M., & Pourmoshtagh, H. (2023). Circular RNAs and inflammation: Epigenetic regulators with diagnostic role. *Pathology Research and Practice*, *251*, 154912. 38238072 10.1016/j.prp.2023.154912

[33] Huang, Y., Xue, Q., Cheng, C., Wang, Y., Wang, X., Chang, J., & Miao, C. (2023). Circular RNA in autoimmune diseases: Special emphasis on regulation mechanism in RA and SLE. *The Journal of Pharmacy and Pharmacology*, *75*(3), 370–384. 36583516 10.1093/jpp/rgac096

[34] Zhang, X., Liu, J., Wang, Y., & Li, H. (2022). Long non-coding RNA NEAT1 promotes inflammatory cytokine production in systemic lupus erythematosus. *Journal of Cellular and Molecular Medicine*, *26*(14), 4032–4044.35726597

[35] Chen, Y., Zhao, H., Wang, L., & Sun, J. (2023). LncRNA NEAT1 promotes macrophage activation and inflammation in rheumatoid arthritis. *International Immunopharmacology*, *117*, 109803.

